# Evaluation of Neurosensory Function Following Inferior Alveolar Nerve Lateralization for Implant Placement

**DOI:** 10.1007/s12663-018-1124-1

**Published:** 2018-06-02

**Authors:** Mukund Rathod, Rajesh Ashok Kshirsagar, Samir Joshi, Sudhir Pawar, Vishal Tapadiya, Suman Gupta, Vrushika Mahajan

**Affiliations:** Department of Oral and Maxillofacial Surgery, Bharati Vidyapeeth Dental College and Hospital, Pune, Maharashtra India

**Keywords:** Inferior alveolar nerve (IAN) lateralization, Neurosensory disturbances, Semmes–Weinstein monofilaments (SWM), Mandibular atropy

## Abstract

**Background:**

Adequate bone height and width is the most important parameter for success of implants. Prolonged edentulous area in mandibular posterior region is often associated with atrophy precluding the use of dental implants. Inferior alveolar nerve (IAN) lateralization is a challenging surgical procedure as it involves the exposure of the neurovascular bundle from its compact bony compartment and adequate retraction while immediate placement of implant.

**Aim:**

Evaluation of neurosensory disturbances related to IAN lateralization for implant placement in the posterior atrophic edentulous mandible.

**Materials and Methods:**

Ten patients above the age of 18 years with an edentulous span in mandibular posterior region showing distance from alveolar crest to IAN ≤ 8 mm (CBCT) were included in the study. The postoperative analysis of NDs was done using Semmes–Weinstein Monofilaments (SWM). Readings were made on the 1st and 7th postoperative day and every month thereafter until the neural sensations were restored.

**Results:**

All patients reported neurosensory disturbance on post-op day 1. None of the patients responded to SWM lesser than 4.56 on first postoperative day, which indicated 100% incidence of neurosensory disturbances. The minimum time required for complete recovery was 2.0 months, and maximum was 4.0 months.

**Conclusion:**

IAN lateralization is a useful method for managing the atrophic posterior mandible with dental implants. If done precisely with experienced personnel, it can provide a worthy option for surgical restoration of atrophic mandible with minimal temporary NDs.

## Introduction

Mandibular atrophy is a consequence of long-term edentulous span in the lower jaw which often leads to functional and esthetic problems. Problems include insufficient retention of the prosthesis, difficulty in speech and masticatory insufficiency [1]. Missing posterior teeth lead to loss of soft tissue support and loss of vertical dimension. This gives “aged face” look, prognathic appearance, unhappy face appearance and a reduced range of expression.

The prosthetic restoration of the partially edentulous atrophic posterior mandible has proved to be problematic. Historically, for these patients, removable partial denture was the only treatment modality available. This was most often because of lack of adequate ridge height for placing dental implants, thus denying patients a most promising dental rehabilitation option [1–3].

**Fig. 1:**
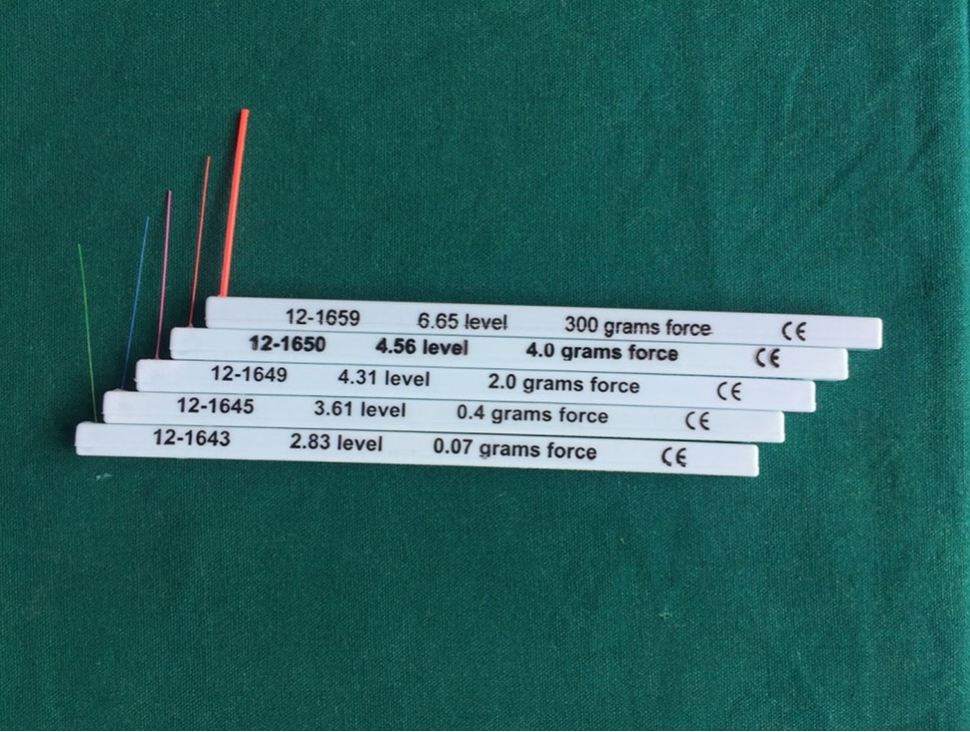
Semmes–Weinstein monofilament

Implant supported prosthesis provide predictable long-term results and deliver a stable functional prosthesis with added advantages of esthetics, increased occlusal force, improved masticatory performance and proprioception (Fig. 1).

Adequate bone height and width is the most important parameter for success of implants. Prolonged edentulous span in mandibular posterior region is often associated with atrophy precluding the use of dental implants [2].

**Fig. 2:**
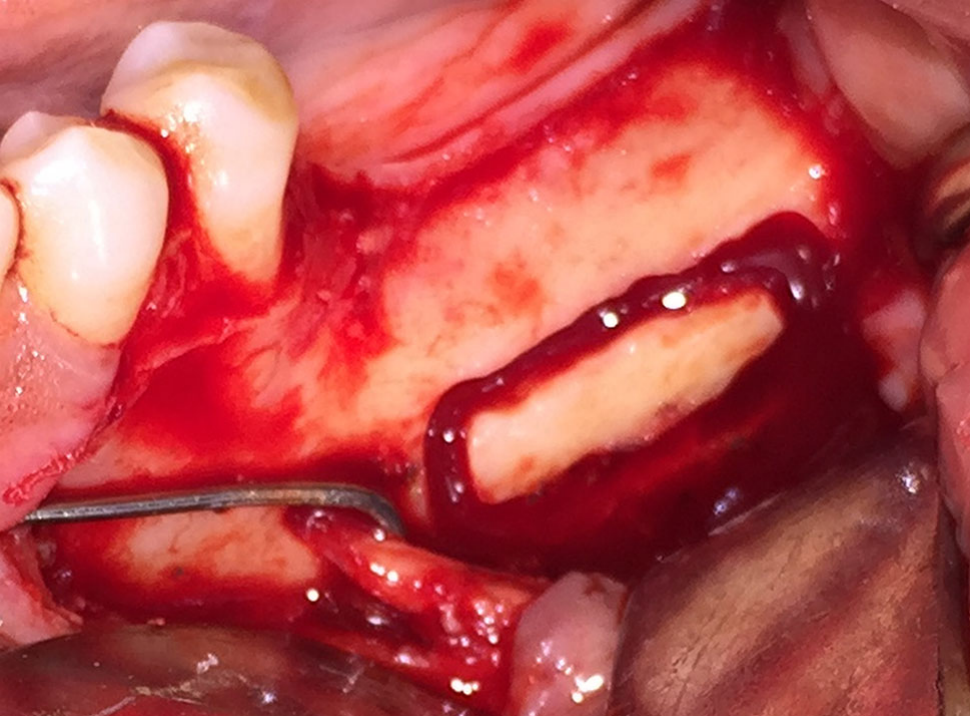
Osteotomy

Inferior alveolar nerve (IAN) lateralization is a challenging surgical procedure as it involves the exposure of the neurovascular bundle from its compact bony compartment and adequate retraction while immediate placement of implant [2, 4–6]. This procedure requires good clinical experience, knowledge of the anatomy and ability to treat potential complications. Neurosensory disturbances (ND) include temporary or permanent anesthesia, paresthesia, dysesthesia and hyperesthesia of the nerve and are the single most important complication of the procedure [7–9]. The risk of fracture of the mandible is also associated due to loss of bone in the atrophied mandible. In previous studies for comparison among surgical techniques of “displacement of the foramen” and the “lateralization of the inferior alveolar nerve,” it has been found that the NDs are more in cases of displacement of the foramen (Fig. 2).

In this prospective study, we have conducted the evaluation of NDs related to IAN lateralization for implant placement in the posterior atrophic edentulous mandible. Pre- and postoperative NDs were evaluated by objective analysis using Semmes–Weinstein monofilaments (SWM).

The previous studies conducted for evaluation of NDs in IAN lateralization for implant placement were subjective in nature [10–12]. They used evaluation technique like questionnaires which may lack objectivity. In addition, this method does not support a scoring system of NDs for better quantification and communication. The SWM provides objective evaluation and eliminates the bias in the results and introduces an ease of quantification, hence using it enhances the communication and comparison between health professionals [3, 13, 14].

## Materials and Methods

From September 2013 to December 2015, ten patients with posterior edentulous areas in the mandible underwent lateralization of the inferior alveolar neurovascular bundle and subsequent implant placement at the Department of Oral and Maxillofacial Surgery, Bharati Vidyapeeth University, Dental College and Hospital, Pune. All patients were assessed for incidence, magnitude and duration required for recovery from NDs using Semmes–Weinstein monofilaments (SWM).

**Fig. 3:**
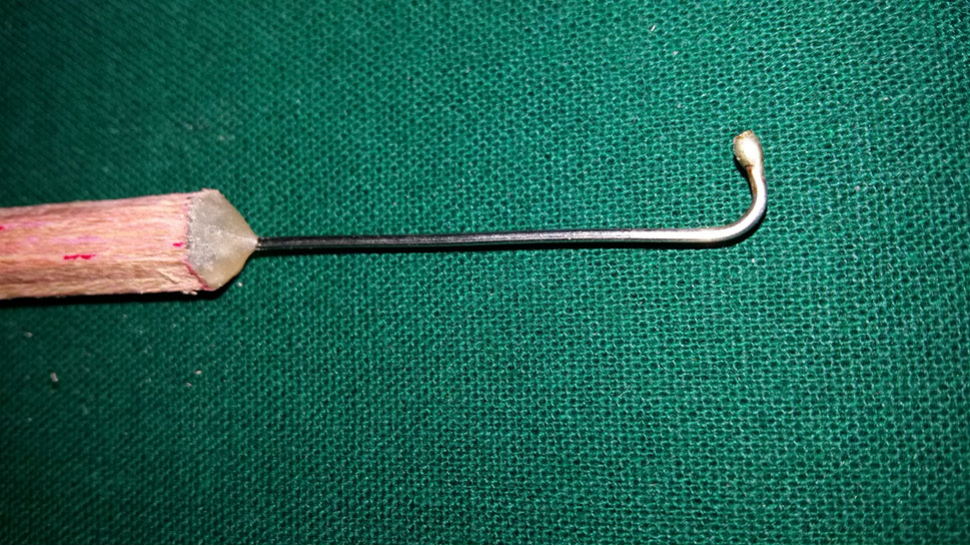
MSR lateralizer

In addition to routine investigations required for surgical intervention, orthopantomogram (OPG) and cone beam computerized tomography scan (CBCT) were carried out for diagnosis and treatment planning (Fig. 3).

This study was approved by the Research Academy and Ethical Committee of the Institute.

**Fig. 4:**
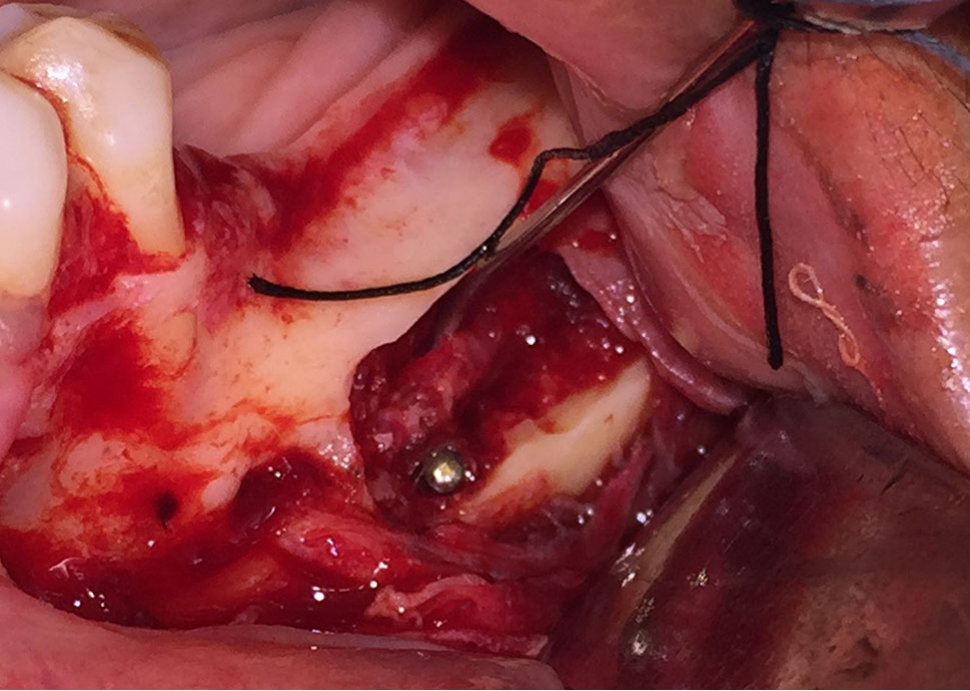
Nerve lateralization

This prospective study included ten patients above the age of 18 years with an edentulous span in mandibular posterior region. It was necessary for the distance from alveolar crest to inferior alveolar nerve ≤ 8 mm (CBCT) to be included in the study. Only patients opting to place dental implant for prosthetic restoration were included. None of the patients had systemic conditions precluding minor oral surgical procedures (Fig. 4).

**Fig. 5:**
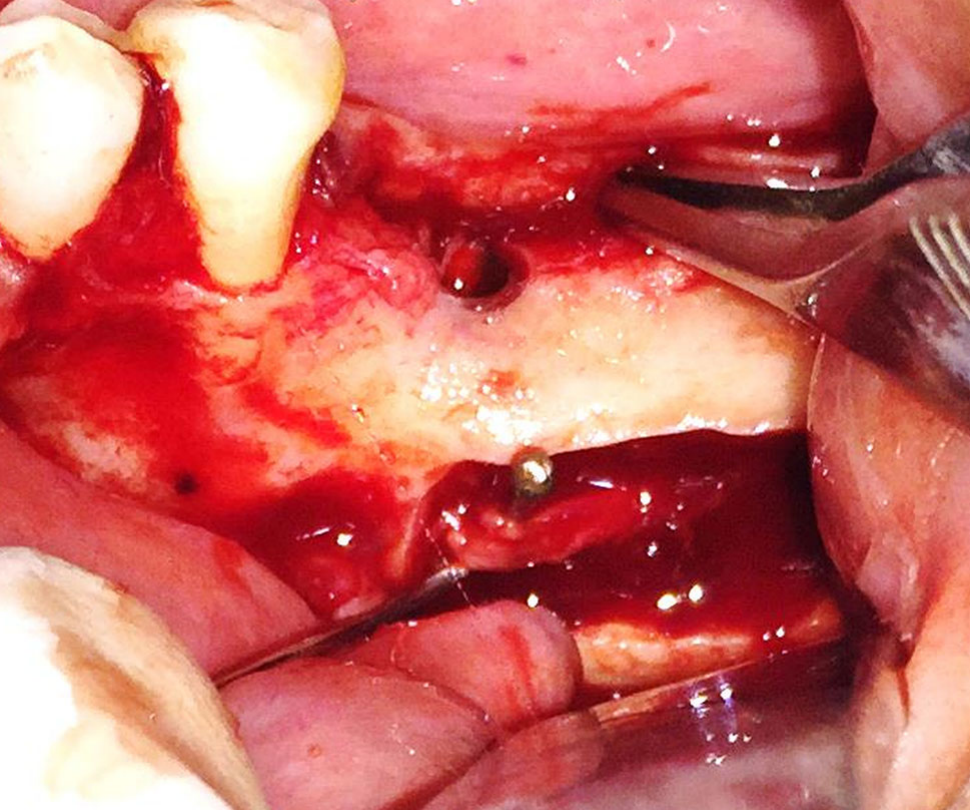
Nerve lateralization 1

Semmes–Weinstein monofilaments are calibrated nylon monofilaments used to measure the patient’s ability to sense a point of pressure. A set of 5 Baseline Tactile SWM Set filaments were used (2.86, 3.61, 4.31, 4.56, and 6.65) for the evaluation. They generate a precise amount of stress over the area of application. The higher the value of the monofilament, the stiffer and more difficult it is to bend. In our study, we used monofilament to evaluate incidence, magnitude and duration of NDs (Fig. 5).

**Fig. 6:**
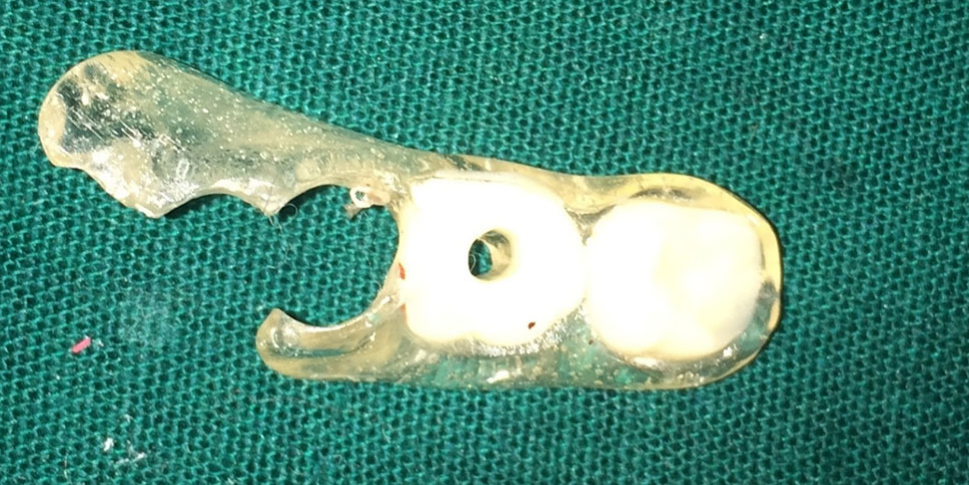
Guiding stent

In the preoperative evaluation procedure, the patient was seated comfortably with his eyes closed to eliminate visual input. The filaments were applied perpendicular to the skin with enough force to cause the monofilament to buckle for approximately 1 s. The evaluation was carried out at predetermined 3 points based on the running course of mental nerve for obtaining standardized and comparable results. Readings were obtained for the proposed surgical side as well as the contralateral side which would serve as a control (Fig. 6).

The surgical procedure was done under local anesthesia (IAN, Lingual and Long buccal nerve blocks). The incision began from the retromolar region and was carried forward to the mesial portion of the cuspid tooth area, where a vertical relaxing incision was made. A full thickness mucoperiosteal flap was reflected. Mental nerve bundle was located and secured using a specially fabricated instrument (nerve retractor).

For the purpose of IAN lateralization, the corticotomy marking was done. The corticotomy started 3–4 mm distal to the mental foramen and extended in a distal direction, 1.5–2 cm distal to the provisional implant position. A small round bur in a straight hand piece with high torque and copious amount of sterile isotonic saline irrigation was used to prepare the corticotomy site. Only hand instruments such as small curettes and spoon excavators were used to remove the trabecular bone and gain access to the neurovascular bundle. The IAN was mobilized from its position and released from the canal using a specially fabricated instrument. After the complete release from the canal, the inferior alveolar nerve was lateralized completely and held in position till accomplishment of immediate implant placement.

Pilot drill was used to determine desired location of the implant placement using a prefabricated stent. After completion of the osteotomy with last implant drill, the implant was inserted while protecting the nerve bundle. Implant of longer length (11.5–13 mm) was inserted ensuring that the apical ends of the implants were positioned inferior to the canal. Once the implant was in its final position, the nerve was left back over the lateral aspect of the implant. The autogenous bone-graft obtained from the corticotomy was mixed with Tricalcium phosphate hydroxyapatite (TCP-HA) crystals and placed at the osteotomy site to fill the defect. Suturing was done to achieve primary closure.

The suitable medications were prescribed to the patient. Methylcobalamin was prescribed to the patient for 4 weeks, although its usefulness in nerve recovery is debatable. Patient was recalled on the first postoperative day, and radiographs were made.

The postoperative analysis of NDs was done using SWM filaments as described earlier. Readings were made on the 1st and 7th postoperative day and every month thereafter until the neural sensations were restored.

## Results

A total of ten patients underwent IAN lateralization for implant placement in the mandibular posterior edentulous region under local anesthesia and sedation. All patients were evaluated for incidence, magnitude and duration of recovery from surgically induced neurosensory disturbances using Semmes–Weinstein monofilaments. All patients were available for the duration of the study.

### Incidence

All patients reported neurosensory disturbance on post-op day 1. None of the patients responded to SWM lesser than 4.56 on first postoperative day, which indicates 100% incidence of neurosensory disturbance (Table 1) (*n* = 10–100.0%).

**Table 1:** Bell’s interpretation scale

Group	Bell’s interpretation scale	Manufacturer’s marking (Fmg)	Calculated force (gm)	Calculated stress (gm/sq mm)
Group A	Normal	2.83	0.0045–0.07	1.45–4.83
Group B	Diminished light touch	3.22–3.61	0.166–0.4	11.1–17.7
Group C	Diminished protective sensation	3.84–4.31	0.692–2	19.3–33.1
Group D	Loss of protective sensation	4.56–6.65	4–300	47.3–300
Group E	Unstable	6.65-more	300 or more	300 or more

### Magnitude

The mean value for magnitude of ND on first postoperative day was found to be 4.33 with standard deviation of ± 0.079. The minimum value on day 1 was 4.31, and maximum was 4.56. At the completion of one week, the minimum value recorded was 3.61 and maximum value recorded was 4.56.

### Duration

The average time for complete recovery from NDs was. The median time to full neurosensory recovery was 3 months. The minimum time required for complete recovery was 2.0 months, and maximum was 4.0 months. The maximum follow-up required was 4 months.

## Discussion

Implants help us to overcome most problems associated with traditional prosthesis. However, in patients with atrophied mandibles, sufficiently long implant fixtures cannot be placed because of potential risk of injury to the inferior alveolar nerve (IAN).

If the distance between alveolar crest and superior border of the inferior alveolar canal is less than 8 mm, it is recommended to carry out lateralization of inferior alveolar nerve to permit placement of longer implants [4].

For IAN lateralization, the IAN is exposed and it is retracted laterally during the implant placement. Following implant placement, the nerve is repositioned over the lateral aspect of the implant. In this technique, there is direct handling of the nerve. This commonly leads to temporary neurosensory disturbances in the region supplied by the nerve in the lower lip and chin area. It would be prudent to note that this study attempts to evaluate neurosensory disturbances following inferior alveolar nerve lateralization and does not deal with the success of the implants or their prosthesis [15–17].

In this prospective clinical study, the incidence, magnitude and duration required for recovery from neurosensory disturbances were evaluated.

The incidence, magnitude and duration of neurosensory disturbances were evaluated by the light touch test using Semmes–Weinstein monofilaments (SWM) at predetermined intervals of time. Neurosensory evaluation was carried out on 1st and 7th postoperative day and every month thereafter until up to such a point where the nerve recovery was complete.

## Incidence of Neurosensory Disturbances

Hashemi [18] evaluated NDs in 82 patients at 110 sites who were treated for IAN lateralization for implant placement. His patients noted NDs in the first week, but the sites with NDs decreased to 29 (26%) at the end of the first month. This reduced to three sites (3%) at the end of the sixth month with no changes from then to the end of the year.

Morrison et al. [19] performed 26 IAN transpositions in 15 patients from 1994 to 1999. After the subjective and objective assessment of neurosensory function author stated that; all the patients reported initial change in the sensation lasting approximately for one month.

The interesting retrospective study by Kan et al. is the only one that compares both surgical techniques of “displacement of the foramen” and the “lateralization of the inferior alveolar nerve”. He analyzed 21 surgeries (64 implantations) after 10–67 months. He found out that sensory disorders occurred significantly more often in cases of displacement of the foramen (66.7%) compared to the lateralization of the nerve (33.3%) [20].

Diaz performed nineteen IAN lateralization procedures on 15 patients using piezotome. He observed that all of the patients (100%) experienced some numbness during the first week after surgery [1].

Our observations are in accordance with the observation of Hashemi et al., Hirsch, Morrison, Kan et al., Lorean et al., Daiz, Vetromilla et al. [1, 18–23] as there is 100% incidence of neurosensory disturbance on the first postoperative day. Our observations are in accordance with the observation of earlier authors who stated the 100% incidence of NDs. We would like to emphasize that in our objective evaluation, we have found that on the first postoperative day there was diminished sensation or loss of sensation in all patients as per Bell’s interpretation scale.

## Magnitude of Neurosensory Disturbance

We have done an objective evaluation for neurosensory disturbance using Semmes–Weinstein monofilaments. These monofilaments consist of unique markings, individually calibrated to deliver its targeted force within a 5% standard deviation. In our review of literature, no study could be found using monofilaments as an evaluating device to assess the magnitude of the NDs.

Hashemi evaluated NDs in all 110 sites in 82 patients who were treated for IAN lateralization for implant placement. The NDs was anesthesia in 81 sites, hypoesthesia in nine sites, burning in nine sites, pain in eight sites, pinching in two sites, and tickling in one site. At the end of the first month, NDs disappeared in 81 sites (74%). NDs were hypoesthesia in 12 sites tickling in 8 sites, burning sensation in 5 sites and pain in 4 sites. At the end of the first month, nine sites of hypoesthesia reported in the first week had returned to normal. The most common type of ND was anesthesia (81 sites), and the least common type was pinching. The tickling lasted 12 months in two patients [18].

In our study, the numbers of patients with neurosensory disturbances were 10 (100%) till end of the 1st month; it was 9 patients on 2nd month, 8 patients on 3rd month and remained only 2 patients till 4th month. At the end of 4th month, they both responded to 2.83 no. monofilaments. The maximum and minimum responsive value of SWM in our study was 4.56 and 2.83, respectively.

In our study, we have evaluated neurosensory disturbances using Semmes–Weinstein monofilaments which is an objective method. These calibrated SWM generates a precise force over the application area of the skin. These numerical values make it more appropriate for communication and comparison. The Bell’s interpretation scale makes it easier to aid in its clinical application.

## Recovery of Neurosensory Function

Hirsch et al. in 24 mandibular posterior segments performed evaluation of neurosensory disturbance after Inferior alveolar nerve transposition and lateralization. They found that the mean time to full resolution of sensation as judged subjectively and objectively was 4.7 weeks. The neurosensory disturbance after transposition was 5.7 and 3.8 weeks after lateralization [21].

Zuninga et al. studied 130 patients for IAN and LN (lingual nerve) injuries. They found the median duration of recovery was 11 months for IAN patients and 5.5 months for LN patients [4].

Morrison et al. [19] performed 26 IAN transpositions in 15 patients, and they found the mean time to neurosensory recovery was 16 months (range 6–60 months).

Overall, 68 IAN reposition and 11 nerve transposition procedures were performed by Lorean et al. in 57 patients (46 females and 11 males). The duration of neural disturbances after the surgery ranged from 1 to 6 months. In other cases, short-term transient nerve disturbances were reported (for 0–4 weeks). No permanent neural damage was reported [22].

Diaz et al. published a prospective cohort study of IAN lateralization by piezotome and immediate implant placement. They found that 12 patients (80%) reported no neurosensory disturbance during the first check-up 8 weeks after surgery, 14 patients (93.3% of patients/94.73% of procedures) had no neurosensory disturbance. Only one patient presented hypoesthesia 2 years after the procedure [1].

Khajehahmadi et al. compared effect of nerve lateralization and nerve transposition on intact teeth anterior to the mental foramen. They found the numbness of the lower lip at 1 week after the operation was done in both groups. After 3 months, lip sensation showed normal results in both groups, except for one patient in each group who continued to experience some form of hypoesthesia. This abnormal lip sensation persisted at the 12-month follow-up [24].

Vetromilla et al. did IAN lateralization in 125 patients and IAN transposition in 150 patients. The shortest full neurosensory recovery time was 6 months. The longest mean follow-up time was 49.1 months. At the end of follow-up, 7% of the patients still experienced neurosensory disturbances [23].

In our study, we evaluated 10 cases postoperatively on first day, after one week, then monthly up to such a point where the nerve recovery was complete. The maximum duration required for full recovery of nerve function was 4 months. The mean duration was 3 months with a standard deviation of ± 0.67 months. Our results do not corroborate with observations given by Archie Morrison et al. and Zuninga et al. [5, 19] as in their study they found the mean time to neurosensory recovery was 16 months.

The median time to full recovery was 3 months. First neurosensory recovery was noted in 2nd month. Of the 10 cases, 2 (20.0%) cases required 2-months for full recovery from ND, 6 (60.0%) cases required 3 months for full recovery and 2 (20.0%) cases required 4-months for full recovery from ND. In our study, maximum number of patients (60%) had neurosensory recovery in postoperative 3rd month.

In our study remaining 20% of cases recovered in the 4th month which required follow-up duration of 4 months. This is in agreement with the observations of Hirsh et al., Lorean, Diaz, Khajehahmadi et al., Vetromilla et al. [1, 21–24] who indicated duration of neurosensory recovery within 6 months of surgery. The restoration of neurosensory function in our study was 100%.

## Conclusion

IAN lateralization is a useful method for managing the atrophic posterior mandible with dental implants. If done precisely with experienced personnel, it can provide a worthy option for surgical restoration of atrophic mandible with minimal temporary NDs. The risk of permanent damage of the IAN appears to be small. Nevertheless, the possibility of postoperative NDs associated with the procedure should be informed in detail prior to commencement of the procedure. The incidence of neurosensory disturbances may be further minimized by advances in surgical expertise and improved instrumentation.
